# Principles of designing interpretable optogenetic behavior experiments

**DOI:** 10.1101/lm.038026.114

**Published:** 2015-04

**Authors:** Brian D. Allen, Annabelle C. Singer, Edward S. Boyden

**Affiliations:** 1 Media Lab, Massachusetts Institute of Technology, Cambridge, Massachusetts 02139, USA; 2 McGovern Institute for Brain Research, Massachusetts Institute of Technology, Cambridge, Massachusetts 02139, USA; 3 Departments of Biological Engineering and Brain and Cognitive Sciences, Massachusetts Institute of Technology, Cambridge, Massachusetts 02139, USA

## Abstract

Over the last decade, there has been much excitement about the use of optogenetic tools to test whether specific cells, regions, and projection pathways are necessary or sufficient for initiating, sustaining, or altering behavior. However, the use of such tools can result in side effects that can complicate experimental design or interpretation. The presence of optogenetic proteins in cells, the effects of heat and light, and the activity of specific ions conducted by optogenetic proteins can result in cellular side effects. At the network level, activation or silencing of defined neural populations can alter the physiology of local or distant circuits, sometimes in undesired ways. We discuss how, in order to design interpretable behavioral experiments using optogenetics, one can understand, and control for, these potential confounds.

Optogenetic tools allow for the precise control of the electrical activity of genetically targeted neurons by transporting specific ions into or out of cells in response to light. These tools are light-sensitive proteins known as opsins, which are seven-transmembrane proteins that play photosensory or metabolic roles in species throughout the tree of life (Boyden 2011). These opsins respond to light either by pumping ions into or out of cells (e.g., halorhodopsins pump chloride ions into archaea in response to light; bacteriorhodopsins and archaerhodopsins pump protons out of archaea in response to light), or by opening an ion channel (e.g., channelrhodopsins let cations such as sodium, protons, and calcium into eyespots of algae). By expressing these molecules in specific neurons, regions, or projection pathways, the targeted circuit elements can then be silenced or activated in response to light. Halorhodopsins and archaerhodopsins are commonly used for optical silencing of neural activity with light (Han and Boyden 2007; Zhang et al. 2007a; Chow et al. 2010; Gradinaru et al. 2010; Han et al. 2011; Chuong et al. 2014). Channelrhodopsins are commonly used for optical activation of neural activity with light (Boyden et al. 2005; Nagel et al. 2005; Yizhar et al. 2011; Klapoetke et al. 2014).

These molecules have become widespread in neuroscience for the investigation of how specific neural circuit elements contribute to behavior, and are even being contemplated for therapeutic purposes (Chow and Boyden 2013). This popularity is in part because in mammals the light-absorbing component of optogenetic tools (the chromophore all-trans retinal) is naturally present in the brain and body (Ishizuka et al. 2006). To the end of designing and interpreting behavior experiments using optogenetics, it is important to understand the side effects that these optogenetic proteins can cause in living cells, as well as the effects of heat and light on neural functions, and the biochemical activity of specific ions transported by optogenetic proteins. Additionally, activation or silencing of defined neural populations can result in network-level side effects, for example, through synaptically mediated activation of unanticipated downstream neurons. Here we discuss how these considerations can inform the design and interpretation of behavioral experiments that incorporate optogenetics as a tool.

## Cell-autonomous side effects

### Protein expression

Expressing a protein in a cell can result in side effects in that cell. High levels of expression of any protein can, in principle, adversely affect cell health, and even result in cell death (Liu et al. 1999; Klein et al. 2006). Unfortunately expression levels are difficult to accurately characterize in vivo and thus the exact relationship between expression level and toxicity is often not well understood. Determining whether or not a given level of expression (e.g., as governed by gene dosage, promoter choice, and duration of expression) causes toxicity or other side effects is complicated because such effects may depend on factors including species, brain region, cell type, and age of the animal. High expression of a protein may alter electrophysiology as well as cell health: in studies examining the effects of expressing opsins at high levels in mammalian human embryonic kidney 293 (HEK293) cells in vitro, changes were reported in the capacitance of the membrane (Zimmermann et al. 2008). *Drosophila* have expressed opsins under 20 copies of a conditional enhancer the upstream activating sequence (UAS), which may support a recent report of high light sensitivity of neurons in such flies for optogenetic activation (Klapoetke et al. 2014). As another example, the *Natronomonas pharaonis* halorhodopsin first assessed in neurons as an optogenetic silencer candidate (Han and Boyden 2007; Zhang et al. 2007a) has been efficacious in multiple *Caenorhabditis elegans* studies (e.g., Wen et al. 2012), but in mammals appeared to form aggregates when expressed at high levels in cortical neurons (Zhang et al. 2007a; Gradinaru et al. 2008; Zhao et al. 2008). As a result, other silencers have grown in popularity, including the archaerhodopsin-class silencers (Chow et al. 2010), as well as halorhodopsins with appended trafficking-enhancement sequences (Gradinaru et al. 2010; Chuong et al. 2014). Curiously, one transgenic mouse has been created with the original *N. pharaonis* halorhodopsin in the hypocretin/orexin neurons of the mouse hypothalamus, and no abnormal morphology nor changes in cell electrical properties were noted, despite expression levels strong enough to elicit sleep upon light delivery to these cells (Tsunematsu et al. 2011). As another example, newborn neurons in the adult mouse hippocampus were reported to increase channelrhodopsin expression slowly over 3 mo following neuronal differentiation, a relatively slow timecourse (Toni et al. 2008). Thus, it is possible that some cells, under specific gene expression conditions, may express optogenetic tools better or worse than other cells, even within the same species.

Opsins are often fused to fluorescent proteins to facilitate visualization in opsin-expressing cells. The type of fluorescent protein used can modulate opsin function. For example, in zebrafish, the unaltered *N. pharaonis* halorhodopsin appeared to clump when expressed with the fluorophore mCherry, more than it did when fused to the fluorophore YFP (Arrenberg et al. 2009). This differential effect may have persisted even after appending trafficking sequences (e.g., compare Fig. 1B and Supplemental Fig. S1B of the aforementioned citation). Clumping of a channelrhodopsin fused to mCherry also was apparent in Figure 2E of (Atasoy et al. 2008), suggesting that mCherry may not be an ideal fluorophore for opsin usage. Similarly, transgenic animals expressing channelrhodopsin-2 fused to YFP exhibited greater optical excitability than did those with channelrhodopsin-2 fused to tdTomato (Madisen et al. 2012).

How then does one achieve efficacious and safe expression of an optogenetic tool in a cell type of interest? In mammals, viruses expressing opsins under cell-specific promoters in principle are useful, but many cell-specific promoters are not small enough to fit into a virus. Some commonly used promoters can target neurons (e.g., synapsin-1), or simply express pancellularly (e.g., CAG, EF1α) (Kügler et al. 2003; Dittgen et al. 2004; Betley and Sternson 2011), when used in adeno-associated virus (AAV) or lentivirus, two popular methods of viral gene delivery into the mammalian brain. Complicating attempts to target expression, viruses themselves can have various tropisms, i.e., they can infect some cell types preferentially over others. For example, AAV serotype 2/1 has been reported to preferentially target inhibitory neurons, whereas lentivirus pseudotyped with a glycoprotein from the vesicular stomatitis virus (VSVg) has been reported to preferentially target excitatory neurons (Nathanson et al. 2009). Promoter–virus combinations may thus need to be optimized jointly in order to guarantee a high level of specificity.

Accordingly, many groups working with mammals have turned to transgenic mice, which can be equipped with large promoters inserted, for example, in bacterial artificial chromosome (BAC) form, to enable expression of an opsin in a specific cell class (Arenkiel et al. 2007; Borgius et al. 2010; Zhao et al. 2011). However, in addition to targeting gene expression to specific cells, such large promoters can, in principle, contain DNA sequences that modulate opsin or cellular functions. For example, the choline acetyltransferase (ChAT) gene is expressed in cholinergic neurons, and its promoter has been used to make transgenic mice expressing channelrhodopsin-2 in cholinergic neurons. This promoter, however, contains an entire gene which happens to encode for the vesicular acetylcholine transporter (VAChT), and mice thus created exhibit altered cholinergic tone and concomitant changes in behavior (Kolisnyk et al. 2013).

A hybrid method achieves targeted gene expression by taking advantage of the many hundreds of mice that express Cre recombinase transgenically under a specific promoter (via BAC transgenesis, knock-in, or other methods; see databases by the GENSAT project (Gong et al. 2003, 2007; web site: www.gensat.org; www.ncbi.nlm.nih.gov/projects/gensat) or Allen Mouse Brain Connectivity Atlas (Oh et al. 2014; web site: 2014 Allen Institute for Brain Science. Allen Mouse Brain Connectivity Atlas [Internet]. Available from: http://connectivity.brain-map.org/). The opsin is then delivered via a virus that infects broad sets of cells but whose gene expression is gated by Cre recombinase expression in the target cells. The gene of interest is flanked by lox sites organized so that Cre causes the deletion of a stop sequence or reversal of the gene sequence from a backward-to-forward direction (Atasoy et al. 2008; Kuhlman and Huang 2008; Sohal et al. 2009). Alternatively, such Cre mice can be bred with transgenic mice whose genomes code for opsins in similar Cre-dependent expression cassettes, so that offspring selectively express the opsin in defined sets of cells (Madisen et al. 2012). When using such mice it is important to check for “leaky” or non-Cre-dependent expression. For instance transient expression of Cre early in life could cause recombination that will then persist throughout life, causing enduring, and perhaps undesired, opsin expression in those cells (Betz et al. 1996; Metzger and Feil 1999; Zhuang et al. 2005).

In *Drosophila* (Schroll et al. 2006; Zhang et al. 2007b) and zebrafish (Douglass et al. 2008; Baier and Scott 2009), the GAL4/UAS expression system is commonly used because animals expressing GAL4 under a promoter can be crossed with animals with an opsin behind a UAS, enabling targeted expression via pairwise breeding. Of course, transgenic animals with cassettes bearing opsins under specific promoters can also be directly generated, e.g., in *C. elegans* (Nagel et al. 2005). Other expression systems—for instance the Tet off system (Zhu et al. 2009; Bundschuh et al. 2012), the Cre/lox (see above) or FLP/FRT systems (Schmitt et al. 2012; Fenno et al. 2014)—can also be used in many different species.

Optimizing the expression of opsins in the targeted cell type early in an experiment can save time and effort later on. When viruses are used, it is helpful to determine which virus produces optimal expression (in terms of volume of tissue infected, density of infected cells, and amplitude of expression in cells) empirically; one can obtain aliquots of viruses with many different promoters and serotypes (i.e., from a core facility), and test them in parallel in mice, evaluating the outcome using histology. For example, we often try multiple AAV serotypes, e.g., 8 and 5, as well as 2, 9, and dj (if desired), varying the concentration by diluting virus down from its source concentration (e.g., 5×, 10×, etc. dilutions from the original concentration), in order to identify a viral dose that maximizes the number of target neurons infected, without resulting in overexpression within individual cells. When using viruses to infect cells, the proportion of target cells that express the opsin will vary from animal to animal. This variability can be harnessed for scientific discovery by using it to create a dose–response curve in which the proportion of cells infected is the dose (e.g., Aponte et al. 2011; Kim et al. 2012). Strategies to achieve a high proportion of opsin-expressing cells will vary depending on the opsin, cell type, and species; therefore we recommend trying multiple approaches in parallel as described above. Physiological validation is important, ideally in the context of the actual experiment. For example, in a study in zebrafish cardiology, investigators observed that halorhodopsin expression in cardiomyocytes varied among transgenic zebrafish. They screened for animals with strongest light responses, which occurred in about a quarter of the fish (Arrenberg et al. 2010), which facilitated later experimentation.

The time that opsins take to express can vary depending on the species, age, cell type, mode of delivery, or cell compartment desired for photoperturbation. Thus, empirical determination of expression at different time points after initiation of expression may be of use. For example, it is not uncommon to perform experiments with mice 4 wk after viral delivery (e.g., by AAV), but many groups wait six or more weeks for opsin expression to reach distal axons of an infected cell (Ciocchi et al. 2010; Witten et al. 2010; Tye et al. 2011; Felix-Ortiz et al. 2013; Kim et al. 2013), though in cholinergic neurons virally infected at a young age, just 3 wk were reported to be effective for axonal expression (Kalmbach et al. 2012).

Given the potential for side effects of expressing opsins in cells, assessing toxicity may be of utility for specific scientific or preclinical questions, and can be aided by analyzing cells with respect to accepted criteria or markers of toxicity for the cell type of interest (Gradinaru et al. 2007; Han et al. 2009; Doroudchi et al. 2011; Miyashita et al. 2013). Experiments to assess toxicity ideally should insure that an opsin (or its attached fluorophore) has not adversely altered gross cell morphology or function, and may include histology, electrical recordings, molecular profiling, and other strategies. To address “leaky” expression of opsins, e.g., in cell types other than the one targeted, one can assess whether the levels of expression in nontargeted cells are meaningful, for instance whether the undesired cells express significant amounts of protein, or respond to light stimulation (Madisen et al. 2012). Experiment design should enable comparison of results obtained from both opsin-infected and control cells or animals. Controls include tissue infected with viruses bearing a fluorescent protein with no opsin fused (though such viruses will express cytosolically, rather than near the membrane as if anchored to an opsin), or potentially an opsin with residues mutated to prevent light-activation but with otherwise normal folding and trafficking.

### Heat and light

Light, when absorbed by brain tissue, results in heating. The exact degree of heating for a given wavelength, duration, and power of light delivered depends on intrinsic brain properties, such as the local volume and rate of blood flow, as well as extrinsic properties, such as whether the brain is exposed to ambient temperatures after skull removal, which may encourage cooling (Kalmbach and Waters 2012). Some reports have indicated that powers of light commonly used in optogenetics may result in heating of a fraction of a degree or more, even with modest pulse durations and frequencies (Elias et al. 1987; Christie et al. 2013). Heating of cells has been shown to increase activity of specific kinds of neurons in multiple species (Moser et al. 1993; Long and Fee 2008), although neurons equipped with different channels or receptors may undergo different kinds of changes in response to heating. Light has been shown to result in artifactual fMRI signals in the BOLD response, when delivered to the brain (Desai et al. 2011; Christie et al. 2013), perhaps due to heating.

Light can also have nonthermal effects on cells: light-sensitive molecular pathways (opsin-mediated or otherwise) naturally found in optogenetically targeted cells or their neighbors could potentially cause undesired effects. For example, in *Drosophila* and *C. elegans*, light has been shown to drive cellular signaling via newly discovered signaling pathways (Edwards et al. 2008; Liu et al. 2010; Xiang et al. 2010). In *C. elegans*, reactive oxygen species driven by illumination have been implicated in this signaling (Bhatla and Horvitz 2015). Light-receptive molecules have been reported to exist in brains of the vertebrates (Okano et al. 1994; Blackshaw and Snyder 1999; Koyanagi et al. 2013). Light may have other effects on experiments, such as causing photoelectric artifacts on neural recording electrodes (Ayling et al. 2009; Han et al. 2009). Light may also distract the animal, perhaps eliciting increased locomotion (Godsil and Fanselow 2004) or encouraging the use of a visual distractor to mask the optogenetic stimulus (Huber et al. 2008). Choosing wavelengths of light that the animal cannot see, or is less responsive to, may ameliorate this (Inada et al. 2011; Klapoetke et al. 2014), as can usage of animals that are engineered to be insensitive to light (Kocabas et al. 2012).

To address the potential effects of light and heat, optogenetic experimental design should include controls in which light is delivered in the same manner as the experimental condition but on tissue that expresses a control protein that does not result in neurons responding to light (e.g., with a fluorescent protein, as described above). As red light-sensitive molecules come into use (Chuong et al. 2014; Klapoetke et al. 2014), in some species and contexts red light will incur less tissue absorption than blue or green, and thus incur less heating. If only a small section of tissue needs to be illuminated, the use of smaller light sources, lower light intensities, and/or shielding (e.g., in the form of beveled guide cannulas) could be used to limit light delivery to the targeted volume (Zorzos et al. 2010; Tye et al. 2011). Alternately, if a large area must be illuminated, strategies that can reduce unwanted heating include using larger fibers (Goshen et al. 2011), fiber tips or lenses that spread light, or multiple light sources (Bernstein and Boyden 2011). Finally, while optogenetic approaches are well suited to manipulate neural activity with millisecond precision, for long-duration experiments that do not require millisecond precision either switching opsins (e.g., step-function opsins [Berndt et al. 2009; Bamann et al. 2010; Yizhar et al. 2011]), or chemogenetic or pharmacogenetic approaches (i.e., using a heterologous receptor and a chemical agonist for that receptor [Shapiro et al. 2012]) might be well worth exploring.

Although not specific to optogenetics, the need for implants that go into the brain is also worth considering when designing an experiment. Devices to deliver light, like implanted optical fibers aimed at deep targets, can displace or damage brain tissue. This may be particularly crucial if regions overlying a region of interest are themselves involved in the behavior, or if scientific questions (e.g., development, dendritic spine plasticity, disease progression) are altered by the presence of an invasive element (Chuong et al. 2014). Recently developed opsins that are sensitive to red light (e.g., the channelrhodopsin ChrimsonR [Klapoetke et al. 2014] and the halorhodopsin Jaws [Chuong et al. 2014]) can alleviate these issues by reducing reliance upon implanted light sources. Because red light can more readily travel through brain tissue, light delivery devices can be placed farther from neurons expressing these red-shifted opsins, in some cases even outside the brain entirely.

### Channel and pump activity

There are many ways in which opsin-mediated neural events may potentially differ from those mediated by natural input. Compared with electrical stimulation, many opsins conduct for longer periods of time, due to the intrinsic kinetics of the channels. Slow depolarizations resulting from optogenetic drive may engage endogenous ion channels, resulting for example in calcium entry through voltage-gated calcium channels (Zhang and Oertner 2006). Prolonged channelrhodopsin-mediated depolarization can also result in depolarization block, a phenomenon in which pulses of light of sufficient duration inhibit action potential firing by causing sodium channel inactivation (Herman et al. 2014). Conversely, the hyperpolarization mediated by silencing opsins can engage hyperpolarization-activated channels (e.g., I_h_ nonspecific cation channels, T-type calcium channels), resulting sometimes in “rebound” action potentials (seen in many papers using either chloride pumps, proton pumps, or electrical stimulation for hyperpolarization, e.g., Madisen et al. 2012), which can be reduced by sculpting the waveform of the light so that such channels are not engaged (Chuong et al. 2014). All opsins exhibit some degree of desensitization during long periods of illumination, which is a function of the brightness and color of the light delivered; it is important to validate, especially for long duration light stimuli, the efficacy of the opsin throughout the experimental timecourse.

Beyond direct voltage driven effects, the specific ions conducted through optogenetic tools may alter neural functions via their interactions with signaling molecules. For example, channelrhodopsins pass multiple species of cation, chiefly protons and sodium, but also calcium and potassium (Nagel et al. 2003). Halorhodopsins and archaerhodopsins actively pump chloride into cells (Han and Boyden 2007; Zhang et al. 2007a) and protons out of cells (Chow et al. 2010), respectively. All of these ions in principle can modulate cellular functions. Calcium has a signaling role in many aspects of cellular physiology, ranging from synaptic plasticity to apoptosis to gene expression (Berridge et al. 2000). It has been reported that channelrhodopsin-mediated calcium entry (using the molecule CatCh, which was engineered toward the goal of higher calcium flux [Kleinlogel et al. 2011]) through the plasma membrane enhances the function of voltage-gated sodium channels, and thus increases neural excitability. Halorhodopsins can pump enough chloride into cells to alter the reversal potential of GABA receptors (Raimondo et al. 2012). Channelrhodopsins expressed in Bergmann glia in cerebellar slices, upon illumination, drive glutamate release, most likely through intracellular acidification (Beppu et al. 2014). In the same preparation, light-driven proton pumps expressed in Bergmann glia could prevent ischemic brain damage, perhaps due to alkalinization of the cells and suppression of glutamate release.

Opsins may also be able to exert their voltage-dependent and ion-dependent signals in intracellular organelles, whether naturally or deliberately targeted there. Glial cells in culture or in visual cortex slices, expressing channelrhodopsin-2, release calcium from intracellular stores upon illumination (Figueiredo et al. 2014; Perea et al. 2014), as evidenced by the blockade of opsin-mediated calcium signals when such stores are depleted with the drug thapsigargin. Deliberately targeting proton pumps to mitochondria of yeast or mammalian cells has been reported to result in cell survival with less reliance on oxidative phosphorylation (Hoffmann et al. 1994; Hara et al. 2013). Thus, optogenetic tools may be utilized to study the effects of specific ions on specific physiological processes. If side effects from the ions conducted by specific opsins are a concern, one useful strategy is to use multiple opsins that result in similar voltage effects, but rely on different ionic species (Adesnik et al. 2012; Stefanik et al. 2012; Kimura et al. 2013).

## Network-level side effects

Because neurons are connected—via synapses, gap junctions, and other interfaces—perturbation of the activity of a cell or set of cells can impact the activity of downstream neurons. While assessing the contribution of a cell or set of cells to network function is of course one of the key uses for optogenetics, activating or silencing a cell or set of cells may cause multiple effects, some of which are not understood or desired. Here we describe some examples of potentially unintended network side effects of the optical perturbation of a set of cells.

On short timescales, manipulating neural activity can lead to network effects like altering the balance of excitation and inhibition. For example, silencing a set of tonically active neurons may deprive downstream regions of input necessary to keep their basal activity levels within the normal dynamic range of operation (e.g., Bao et al. 2002). Experimentally activating or silencing a set of neurons may result in downstream circuits being driven or deprived of activity in ways that do not occur endogenously. This is especially true if an entire population is being synchronously activated or silenced. Activating a set of excitatory neurons could recruit inhibitory neurons that then silence excitatory neurons, perhaps even some of the same ones that were excited by light (Han et al. 2009). Optogenetic silencing of excitatory neurons that drive inhibitory neurons could result in disinhibition of the neurons targeted by the inhibitory neurons (Han et al. 2011), with analogous possibilities for the case of silencing inhibitory neurons (Witten et al. 2010). While these phenomena reflect the action of circuits that exist in a normally functioning brain, they should be taken into account when interpreting the behavioral impact of an optogenetic perturbation.

Plasticity and homeostatic adaptation can also result from perturbations of neural activity. Long-term potentiation, an increase in the synaptic efficacy between neurons, may be induced with presynaptic excitation and postsynaptic channelrhodopsin activation through as few as 20 pairings (Zhang and Oertner 2006). Other changes—including homeostatic plasticity (Turrigiano et al. 1998; Borodinsky et al. 2004; Thiagarajan et al. 2005; Hnasko and Edwards 2012), changes in synaptic function due to retrograde cannabinoid signaling (Kreitzer and Regehr 2001; Wilson and Nicoll 2001), nitric oxide signaling (Lev-Ram et al. 1995), and many other mechanisms that can cause side effects may also be engaged, since they are downstream of neural activation. As described above, these phenomena may be either bugs or features in experiments, depending on how they are interpreted and whether they can reveal new findings in a well-controlled way. For example, optogenetic silencing of the CA1 region of the hippocampus for 30 min can lead to recruitment of another brain region—the anterior cingulate—to help animals remember what contexts to fear, though such recruitment of the anterior cingulate does not occur with brief optogenetic silencing (Goshen et al. 2011), potentially implicating a role for network homeostatic plasticity in memory.

Even over short timescales, optogenetic perturbation can cause recruitment of undesired targets if the photostimulated neurons project to multiple regions. Optogenetic stimulation of axons projecting to one region may result in action potentials that backpropagate to the cell body and also end up driving other, undesired regions. Careful tracing of the anatomy of the cells of the targeted axons, i.e., through retrograde viral techniques, may alleviate this concern if it reveals that the neurons in question project only to the desired region (Betley et al. 2013; Stamatakis et al. 2013). When this approach is either untenable or reveals collateral axons to multiple brain regions, one can silence the cell body (e.g., pharmacologically) while stimulating the axon terminals (Stuber et al. 2011), but of course this also alters any computations that depend on the cell body. One can also infuse the target region of the projection with a neurotransmitter antagonist, to test whether the behavioral effect of optogenetic stimulation of the projections is abolished (Tye et al. 2011). The multiplexed usage of single neurons to control multiple targets has behavioral as well as physiological consequences. For example, inhibition of serotonin neurons or Purkinje cell activity via optogenetic or chemogenetic means can alter core body temperature or blood pressure (Ray et al. 2011; Tsubota et al. 2012), revealing new functions not widely thought of as being associated with these cell types.

To monitor network effects of optogenetic manipulation, careful observation of network activity is crucial. Neural recordings can confirm that optogenetic manipulations are altering activity as intended or can reveal how that activity is altered in unexpected ways. Ideally one would record all possible effects, from changes in intracellular signaling to changes in spike output, in all cells in the nervous system (Marblestone et al. 2013). While this is not currently possible, the development of new technologies, from high-density neural recordings (Berényi et al. 2014; Scholvin et al. 2015) to genetic reporters of activity (Chen et al. 2013) to new microscopy methods (Prevedel et al. 2014), may help us approach this ideal. In addition, optogenetic manipulations that are even more precise, from opsins that target specific cell compartments to light delivery systems that drive more naturalistic patterns of activity, may both reduce unintended network side effects and reveal how more specific or complex patterns of activity alter the network. For instance, two-photon holographic excitation of opsin-expressing neurons may support manipulation of activity with near-single cell resolution (Andrasfalvy et al. 2010; Papagiakoumou et al. 2010; Packer et al. 2012, 2015). In sum, while optogenetic tools allow for the temporally and spatially precise recruitment of well-defined sets of cells, these tools may lead to potential confounds due to protein expression, ions conducted, heat and light, and network effects. Many of these concerns also apply to other causal technologies, such as chemogenetic inhibition of neurons, pharmacology, and electrical stimulation. Here we have discussed both potential confounds of optogenetic methods and several approaches for addressing these confounds using currently available methods. Future developments will allow for more precise and complex manipulations and observations of neural circuits.
